# L-Lactic Acid Production by *Lactobacillus rhamnosus* ATCC 10863

**DOI:** 10.1155/2015/501029

**Published:** 2015-04-01

**Authors:** Ana Lívia Chemeli Senedese, Rubens Maciel Filho, Maria Regina Wolf Maciel

**Affiliations:** Laboratory of Optimization, Design and Advanced Control, Department of Product and Processes Development, School of Chemical Engineering, State University of Campinas, Rua Albert Einstein, 500 Cidade Universitária Zeferino Vaz, 13083-852 Campinas, SP, Brazil

## Abstract

Lactic acid has been shown to have the most promising application in biomaterials as poly(lactic acid). *L. rhamnosus* ATCC 10863 that produces L-lactic acid was used to perform the fermentation and molasses was used as substrate. A solution containing 27.6 g/L of sucrose (main composition of molasses) and 3.0 g/L of yeast extract was prepared, considering the final volume of 3,571 mL (14.0% (v/v) inoculum). Batch and fed batch fermentations were performed with temperature of 43.4°C and pH of 5.0. At the fed batch, three molasses feed were applied at 12, 24, and 36 hours. Samples were taken every two hours and the amounts of lactic acid, sucrose, glucose, and fructose were determined by HPLC. The sucrose was barely consumed at both processes; otherwise the glucose and fructose were almost entirely consumed. 16.5 g/L of lactic acid was produced at batch and 22.0 g/L at fed batch. Considering that lactic acid was produced due to the low concentration of the well consumed sugars, the final amount was considerable. The cell growth was checked and no substrate inhibition was observed. A sucrose molasses hydrolysis is suggested to better avail the molasses fermentation with this strain, surely increasing the L-lactic acid.

## 1. Introduction

The use of biotechnology in sustainable production of chemicals from renewable resources is a practice already considered necessary due to their potential in obtaining products of high added value by making use of renewable and relatively low environmental impact.

The lactic acid is a multifunctional valuable organic acid and recently has been shown to have the most promising application in manufacturing of biodegradable and biocompatible polymers such as poly(lactic acid) (PLA), an ecologically correct alternative for conventional nonbiodegradable plastics derived from petrochemicals [1–4]. Additionally it has been seen as a potentially suitable feedstock for biomaterial with specific desired properties achieved depending upon the raw material and manufacture process used [5]. The poly(L-lactic acid) (PLLA), a biocompatible and biodegradable polymer, has been used extensively for biomedical applications as bone repair [6], surgical meshes, sutures, artificial tissues [7, 8], and scaffolds [9, 10].

Lactic acid fermentation is relatively fast, has high yields, and can lead, selectively, to one of its two stereoisomers or the racemic mixture [11]. This is of relevant significance since it has impact on the polymer properties for biomedical applications.

Most studies conducted on lactic acid production performed their experiments in simple batch system [12], emphasizing the importance of fermentation tests in other modes of operation, such as repeated batch and continuous system with recycle cells. The advantage of batch fermentation is potentially to obtain good yields of lactic acid due to the complete utilization of the substrate and is advised if the cost of this substrate is high. On the other hand, continuous processes have higher volumetric productivity, due to the high dilution rate, and keep the process over longer periods [13].

The use of alternative substrates in fermentation processes, aiming at the utilization of agricultural low cost raw materials or by-products from various industries (molasses, bran, corn syrup, whey, etc.), lowers the cost of the culture medium used and hence the final product. However, these substrates have complex composition whose exact total is often unknown. In addition to the carbon source and other nutrients, some compounds that may be present or even formed during the process steps, as pretreatment, may be factors capable of inhibiting the growth of microorganisms or prevent the synthesis of the metabolite of interest [14].

The most common used substrate for* Lactobacillus (L.) rhamnosus* ATCC 10863 fermentation for lactic acid production is glucose [15–18], but cellulose [19], lignocellulose [20], and sucrose [21] are also used. Molasses hydrolyzed [22] can as well be used and in this work the lactic acid production with this cheap and green substrate without a pretreatment will be checked.

## 2. Materials and Methods

### 2.1. Strain

The L(+) lactic acid homofermentative strain* L. rhamnosus* ATCC 10863 was obtained at Technology and Research Foundation André Tosello, Campinas, SP.

### 2.2. Preparation of Inoculum and Fermentation Medium

The inoculum was maintained at an agar tube at −5°C after being received. The culture was activated in 5.0 mL of MRS agar sterile tube (Neogen Corporation) maintained in incubator (Quimis) at 37°C for 48 hours. Afterwards, an aliquot was transferred to a sterile tube with 10.0 mL of MRS (Neogen Corporation) that was kept in incubator at 37°C for further 48 hours. Then, 5.0 mL of this inoculum amount was transferred to 45.0 mL of MRS broth in 125.0 mL sterile Erlenmeyer flask and incubated once again at 37°C for 48 hours. Subsequently, this volume was transferred to 450.0 mL of MRS broth, resulting in 500.0 mL, in a 1.0 L Erlenmeyer flask. It was maintained at shaker at 37°C with rotation of 150 rmp for 30 hours to activate the strain before the bioreactor fermentation [5].

The fermentation medium used was sugar cane molasses purchased at Usina Costa Pinto SA, Piracicaba, SP, 2012, and yeast extract diluted in distilled water. As the sucrose is the main sugar in molasses [5], this solution concentration was based on sucrose concentration, considering a 100.0 g/L of sucrose first solution. For this reason, the molasses concentration at this study will be named as sucrose concentration. Therefore, the fermentation solution concentration was 27.6 g/L of sucrose and 3.0 g/L of yeast extract, determined by experimental planning in previous work. The yeast extract is an expensive supplement, although necessary, and even so the literature reports many works using this supplement in lactic acid fermentation, as 5.0 g/L [5], 10.0 g/L [22], 15.0 g/L [16], and 30.0 g/L [21]. The volume prepared was 3,071 mL, resulting in a final volume of 3,571 mL after adding the inoculum medium, maintaining the 14.0% (v/v) inoculum.

### 2.3. Batch and Fed Batch Fermentation Processes

Two fermentation processes were performed, to know batch and fed batch. At the fed batch process, molasses feed were added during the fermentation to increase the final concentration of lactic acid.

A bioreactor (New Brunswick BioFlo 415) with capacity up to 7 L was used. A sterilization procedure was performed for 15 minutes at 121°C with the fermentation medium at the bioreactor before the fermentations started. After cooling, the inoculum was added. The temperature utilized was 43.4°C, the agitation was 200 rmp, and the pH was maintained in 5.0 during the fermentations using a base pump with 4.0 N NaOH. The fermentations were performed under anaerobic conditions.

At the batch process the fermentation was kept until the base pump stopped working, indicating the end of acid production. At the fed batch process three molasses feed were added at 12 hours, 24 hours, and 36 hours, and the fermentation was stopped at 48 h. At both fermentation processes, 4.0 mL samples were taken every 2 hours after the start of fermentation to observe the performance.

The molasses feed volumes were the same as the total samples volume collected until the feeding time, maintaining, in this way, the same total amount of fermentation volume. The concentration determined for the molasses feed was 270 g/L of sucrose, about 10 times more than the initial one.

### 2.4. Analytical Determination

The samples (2.0 mL) collected during fermentation were placed in Eppendorf tubes (duplicate) previously dried at incubator (Labor) at 105°C for 4 hours and weighed to determine cellular concentration by gravimetry. Subsequently the samples were centrifuged at Centrifuge 5810 R (Eppendorf) with 3,000 rpm at 25°C for 15 minutes. Afterward they were filtered with Millipore membrane 0.45 *μ*m pores and diluted with deionized water.

HPLC Infinity 1260 (Agilent Technologies) and the column Aminex HPX-87 H (BIO RAD) were used. The operating conditions were temperature of 25°C, 5.0 mM sulfuric acid as mobile phase, and flow rate of 0.6 mL/min. The mobile phase was vacuum-filtered with Millipore membrane 0.45 *μ*m pores and degassed in an ultrasound bath for 30 minutes to prevent bubble formation, which may damage the fuel injection pump or even produce false peaks while passing through the detector. The detector was a refractive index with temperature of 25°C. The method was calibrated with standard solutions of sucrose, glucose, fructose, and lactic acid.

To determine cellular concentration, the precipitates obtained after samples centrifugation for analytical determination were washed with distilled water and centrifuged twice with the same conditions. Following that, the tubes were dried once again at 105°C for 24 hours and weighted to obtain the dry cellular weight.

## 3. Results and Discussion

The amount of sucrose, glucose, and fructose was measured at HPLC, as well lactic acid concentrations produced during the fermentations, and results are displayed in Table 1 for both processes. At the fed batch fermentation, the samples volume taken until 12 hours was around 25.0 mL, so all the feed volumes were the same, since all had 12-hour gap. The molasses feed sugars concentrations were also checked at HPLC and are exposed in Table 1.

**Figure 1:**
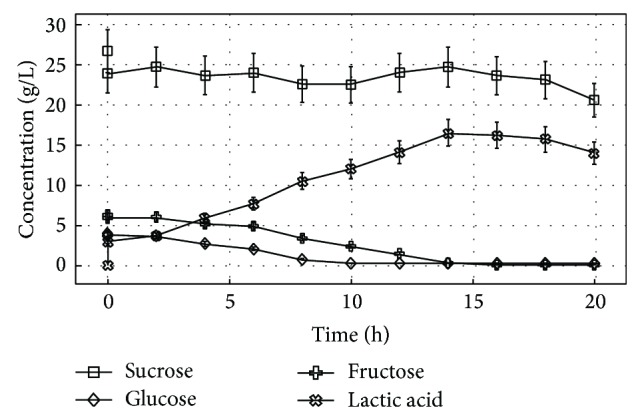
*L. rhamnosus* ATCC 10863 fermentation overview: sugars consumption and lactic acid production at batch fermentation process.

**Figure 2:**
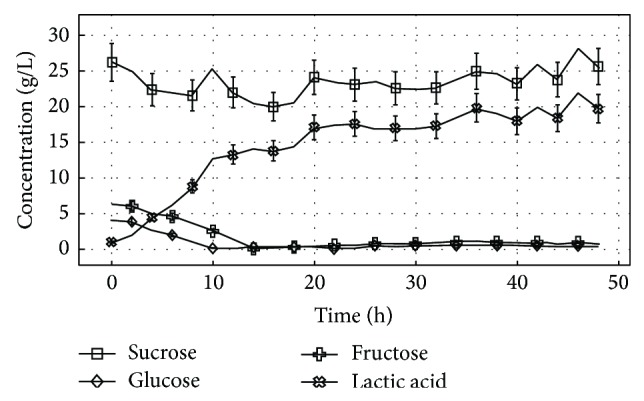
*L. rhamnosus* ATCC 10863 fermentation overview: sugars consumption and lactic acid production at fed batch fermentation process.

At the batch fermentation, the base pump was a constant and straight line at around 18–20 hours, showing the end of process, and the fed batch fermentation was stopped at 48 hours. At the batch fermentation process, it can be observed that the maximum amount of lactic acid produced was at 14 hours, and fed batch process was at 46 hours, with 16.5 g/L and 22.0 g/L, respectively. It can be checked that the sucrose behavior is instable and it was barely consumed at both fermentation processes. Perhaps these concentration oscillations are due to sample collections, experimental and equipment errors. On the other hand, the glucose and fructose were almost all consumed. At batch process glucose was practically zero at 8 hours and fructose 14 hours and at the fed batch process glucose was practically zero at 10 hours and fructose at 14 hours. These sugars remained in very low concentration until the end of both processes. These results can be better visualized in Figure 1 for batch fermentation and in Figure 2 for fed batch fermentation.

At batch fermentation the sucrose behavior was unstable and it is still detected in high amount at the end of processes, maybe due to reasons pointed earlier regarding sucrose concentration oscillations. Otherwise, it was noted that as soon as fructose was completely consumed at 14 hours the production of lactic acid starts to lightly decrease. So, it can be deducted that the increased lactic acid formation during the batch fermentation was due to the glucose and fructose sugars consumption already detected in the medium fermentation since the beginning of process.

At the fed batch fermentation the sucrose behavior was also instable as it was also still detected in higher amount at the process end and this fact cannot be related to the molasses feed, since their concentrations were not high enough to maintain practically the same amount detected in the beginning of fermentation. It is noted that after glucose was over at 10 hours the lactic acid production showed a pattern of increase in a more stable fashion and remained until the end of fermentation, when reaching the maximum production. It is observed in Figure 2 that after each molasses feed the amount of lactic acid quietly increased; however, glucose and fructose were not detected after each feed at HPLC, as observed in Table 1.

The conversions of sugars during the fermentations were calculated individually using the values obtained in Table 1 and (1), considering *C* as conversion rate (%) and *Y* as sucrose, glucose, and fructose (g):

(1) $$\begin{array}{c} C_{Y}=\frac{Y_{0}-Y_{f}}{Y_{0}}\times 100. \end{array}$$

For fed batch fermentation the values taken from Table 1 were *Y*_0_ = 0^∗^ hours and *Y*_*f*_ = 20 hours. The total samples volumes taken during the 20 hours of fermentation were taken into account to calculate the conversion rate at batch process, since the final volume was less than the initial one. Then the conversion rate results for batch fermentation were 14.9% for sucrose, 92.4% for glucose, and 98.5% for fructose. From Table 1, the values for fed batch process calculation of sugars conversion were *Y*_0_ = [0^∗^ hours + 3 × (*F* ^∗∗∗^)] and *Y*_*f*_ = 48 hours. The initial and final fermentation volumes were the same due to the molasses feed applied. As a result, the conversion rate for fed batch fermentations was 38.7% for sucrose, 93.8% for glucose, and 98.3% for fructose.

Srivastava et al. [21] reported a* L. rhamnosus* ATCC 10863 (called* L. casei* subsp.* rhamnosus* NRRL B-445) fermentation at 39°C using sucrose (105.75 g/L) as substrate supplemented with 30.0 g/L of yeast extract and other compounds. The lactic acid production was 80.0 g/L and the batch fermentation time was ~220 hours. It was observed that, until ~40 hours of fermentation, the lactic acid production was ~20.0 g/L and ~60% of initial sucrose was still present at the medium, showing that sucrose was not easily consumed, which can also be observed in the current work. The comparison of the results achieved in this work with those obtained from literature reveals the dependence of the type of bacteria on the sugar conversion and lactic acid production. However, for some specific applications, as is the case for the production of desired isomers for biomedical application, the use of some microorganism even those that lead to lower total conversion is required.

In Yoo et al. [16] work, the* L. rhamnosus* ATCC 10863 (called* L. casei* subsp.* rhamnosus* NRRL B-445) batch fermentation produced 80 g/L of lactic acid using glucose (100.0 g/L) as substrate supplemented with 15.0 g/L of yeast extract and other compounds. If it is taken into consideration that the 16.5 g/L of lactic acid produced at the batch fermentation at the present work was due to the glucose and fructose sugars, since sucrose was not well consumed, the initial sugar can be taken as those sugars sum, that is, 10.3 g/L. Therefore this initial sugar amount is about 10% of the initial sugar amount in Yoo et al. [16] work. Consequently the lactic acid expectation in the present work was supposed to be ~8.2 g/L and it was in fact double this amount, which can be considered as a satisfactory result.

In Aksu and Kutsal [22] work, the* L. rhamnosus* ATCC 10863 (called* L. casei* subsp.* rhamnosus* NRRL B-445) fermentation was performed at 42°C using sugar cane molasses (20.0 g/L). The production yield was 46% and increased to 70% when the substrate was supplemented with 10.0 g/L of yeast extract, 5.0 g/L of peptone, and inorganic salts. The batch fermentation lasted 250 hours. Another fermentation process was performed hydrolyzing the molasses using invertase and the production yield improved to 83%.

**Figure 3:**
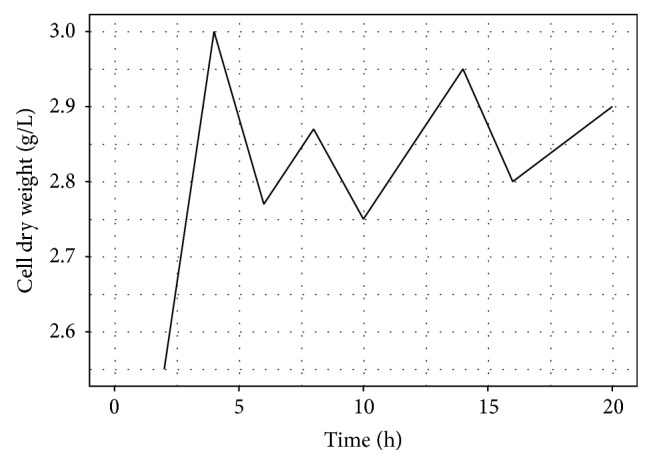
Cell dry weight of* L. rhamnosus* ATCC 10863 at batch fermentation process.

**Figure 4:**
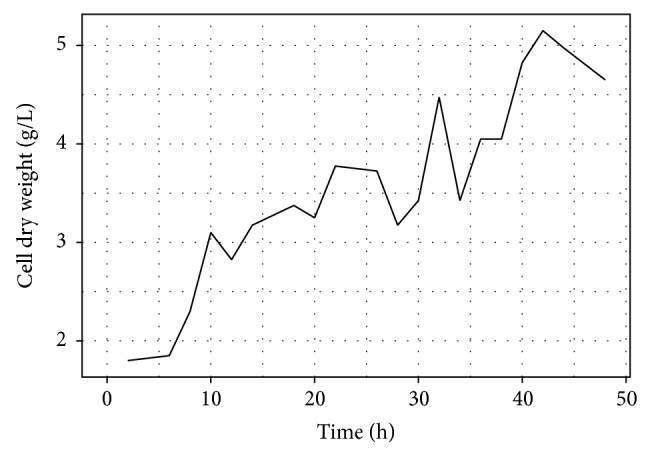
Cell dry weight of* L. rhamnosus* ATCC 10863 at fed batch fermentation process.

The cell growth was investigated in both fermentation processes to check a possible substrate inhibition in the medium.  Figure 3 depicts these results for batch fermentation and Figure 4 for fed batch fermentation.

At the batch process the lag phase was not noticed and the cell growth entered to exponential and remained on a stationary phase after ~6 hours of fermentation. The cell dry weight of was sharply increased to a maximum value of 3.0 g/L at period of 4 hours of fermentation and lactic acid was being produced already. Substrate inhibition was not verified.

At the fed batch process the cell growth presented a lag phase. The log and stationary phase were noted at the first time clearly after the lag phase and again discretely after the three molasses feed at 12, 24, and 36 hours. Subsequently, the decay phase was started at 40 hours. The cell dry weight was sharply increased to a maximum value of 5.1 g/L at a fermentation period of 42 hours, and 4 hours later the lactic acid had the maximum production. No substrate inhibition was observed.

## 4. Conclusions

L-lactic acid was produced in batch and fed batch fermentation processes using the strain* L. rhamnosus* ATCC 10863 and no cell inhibition was detected. 16.5 g/L of lactic acid was produced at the batch and 22.0 g/L at the fed batch. The fed batch strategy produced 33.3% more lactic acid than the batch, taking into account more than double fermentation time. The amount of lactic acid produced in this work can be improved by making use of a pretreatment process of the sugar cane molasses by hydrolysis with invertase, as the substrate has sucrose as its main sugar and this sugar was not significantly consumed when compared to glucose and fructose. One sucrose molecule generates one glucose and one fructose, and knowing that this strain digests these sugars the sugar cane molasses will be availed, reaching the goal of using a cheap and green substrate to obtain a high added value, as the PLLA for posterior biomedical applications. Once this product is a high added value one, the fermentation process costs, including the use of yeast extract as a supplement, are practicable especially when it may be a by-product from the bioethanol production.

## Figures and Tables

**Table 1:** Sugars and lactic acid values obtained at batch and fed batch *L. rhamnosus* ATCC 10863 fermentations.

Time (h)	Batch fermentation				Fed batch fermentation			
	Sucrose (g/L)	Glucose (g/L)	Fructose (g/L)	Lactic acid (g/L)	Sucrose (g/L)	Glucose (g/L)	Fructose (g/L)	Lactic acid (g/L)
0	26.7	4.0	6.3	0.0	26.5	4.1	6.5	0.0
0^∗^	23.9	3.7	5.9	3.0	26.2	4.0	6.3	1.0
2	24.7	3.7	6.0	3.7	25.0	3.9	6.1	2.0
4	23.7	2.7	5.2	5.9	22.4	2.7	5.0	4.5
6	24.0	2.1	4.9	7.7	22.0	2.0	4.6	6.3
8	22.6	0.8	3.4	10.5	21.6	1.1	3.6	8.8
10	22.5	0.3	2.4	12.0	25.4	0.2	2.6	12.9
12^∗∗^	24.0	0.3	1.5	14.1	22.0	0.2	1.4	13.3
14	24.7	0.3	0.3	16.5	20.5	0.3	0.1	14.1
16	23.6	0.3	0.1	16.2	20.0	0.3	0.2	13.8
18	23.1	0.3	0.1	15.7	20.6	0.4	0.4	14.5
20	20.6	0.3	0.1	14.0	24.1	0.4	0.5	17.1
22	—	—	—	—	23.4	0.1	0.6	17.5
24^∗∗^	—	—	—	—	23.1	0.1	0.6	17.6
26	—	—	—	—	23.6	0.5	0.8	17.1
28	—	—	—	—	22.6	0.4	0.8	17.0
30	—	—	—	—	22.5	0.5	0.9	17.1
32	—	—	—	—	22.7	0.5	0.9	17.3
34	—	—	—	—	23.9	0.6	1.1	18.6
36^∗∗^	—	—	—	—	25.0	0.6	1.2	19.8
38	—	—	—	—	24.7	0.6	1.1	19.2
40	—	—	—	—	23.2	0.5	1.0	18.0
42	—	—	—	—	26.0	0.5	1.0	20.1
44	—	—	—	—	23.8	0.4	0.8	18.4
46	—	—	—	—	28.2	0.5	1.0	22.0
48	—	—	—	—	25.6	0.4	0.8	19.7
*F* ^∗∗∗^	—	—	—	—	270.0	35.5	55.0	0.0

^∗^Molasses fermentation medium + inoculum added = fermentation start.

^∗∗^Concentrated molasses feed applied.

^∗∗∗^Molasses feed sugars concentration.
